# Anticancer Potential of Flavonoids: Their Role in Cancer Prevention and Health Benefits

**DOI:** 10.3390/foods13142253

**Published:** 2024-07-17

**Authors:** Yeonhee Pyo, Ki Han Kwon, Yeon Ja Jung

**Affiliations:** 1 Department of Beauty Cosmetics, College of Biomedical and Health Science, Konkuk University, Chungju 27478, Republic of Korea; 2 College of General Education, Kookmin University, Seoul 02707, Republic of Korea; kihan.kwon@kookmin.ac.kr

**Keywords:** flavonoids, apoptosis, bioflavonoid, citrus fruits

## Abstract

The term “flavonoid” encompasses a group of plant compounds, predominantly flavonoids, present in fruits, vegetables, and other plant-based foods. These compounds deliver significant health benefits, including potent antioxidant properties that protect cells from free radicals, thereby mitigating aging and disease. We assessed study quality and bias using the Cochrane Risk of Bias tool and the Newcastle−Ottawa Scale. Inclusion criteria specified that the studies must examine a natural flavonoid from fruits, must involve animal or human trials, must be original studies, and must be English articles on the flavonoid’s health and cancer-prevention effects, excluding conference abstracts and single-case studies. We conducted a comprehensive search of major databases including PubMed, Web of Science, Embase, SCOPUS, and Google Scholar, reviewing six clinical trials with total sample sizes of over 50 to 1500 participants. The results indicate that consuming flavonoid-rich fruits can aid in cancer prevention by targeting angiogenic and cancer-protective pathways. We specifically selected tomatoes, mulberries, Amazon grapes, apples, and citrus fruits due to their well-documented high levels of flavonoids and the robust clinical evidence supporting their physiological effects. In particular, citrus fruits contain additional beneficial phytochemicals that complement the action of flavonoids, enhancing their overall health effects. The anti-cancer mechanisms of flavonoids are not well-defined in the scientific literature, suggesting a gap that this study aims to address. Our study provides novel contributions by demonstrating how flavonoid supplementation induces anti-cancer effects through angiogenesis, anti-inflammatory actions, antioxidant-induced apoptosis, and modulation of pathways like PI3K/Akt and MAPK. These effects were particularly notable in the prevention and progression of breast, colon, liver, and lung cancers, with statistical significance (*p* < 0.05). By elucidating specific mechanisms and pathways, this study contributes to the understanding of flavonoids’ role in cancer prevention and underscores the potential for developing natural anti-cancer therapeutics through the inclusion of flavonoid-rich fruits in the diet. Future research should focus on randomized controlled trials assessing long-term effects of flavonoid supplementation in diverse populations, exploring optimal dosages, and understanding interactions with conventional cancer therapies to provide comprehensive evidence for clinical applications.

## 1. Introduction

The term “flavonoid” refers to a group of plant compounds, primarily flavonoids. Flavonoids can be obtained from a variety of fruits, vegetables, and plant-based foods and have a number of health benefits, including antioxidant effects. Rutin is one of the specific flavonoids found in fruit [1,2]. Flavonoids, sometimes referred to as bioflavonoids, are compounds that are an important component of a healthy diet and offer overall benefits to human health due to their antioxidant, anti-inflammatory, cardiovascular-health-enhancing, immune-system-boosting, and cancer-preventing effects [3]. The effects of flavonoids are very powerful and protect cells from harmful compounds such as free radicals, thereby preventing aging and disease [4]. They also regulate the immunomodulatory system, making them effective for preventing infections and inflammation [5]. They strengthen blood vessels to improve circulation and help prevent cardiovascular disease, especially by preventing damage to blood-vessel walls and reducing inflammation [6]. In addition, studies have shown that they support brain health, even impacting dementia and neurological diseases [7]. Despite the broad spectrum of health benefits, the specific mechanisms through which flavonoids exert their anti-cancer effects remain poorly understood. This study aims to address this gap by investigating how flavonoid-rich fruits contribute to cancer prevention. We focus on specific fruits such as tomatoes, mulberries, Amazon grapes, apples, and citrus fruits, which are known for their high flavonoid content and have robust clinical evidence supporting their health benefits. Citrus fruits, in particular, contain additional beneficial phytochemicals that work synergistically with flavonoids to enhance their overall health effects. Understanding these combined effects is crucial for developing effective dietary strategies for cancer prevention. Our research demonstrates how flavonoid supplementation induces anti-cancer effects through various mechanisms, including regulation of angiogenesis, anti-inflammatory actions, antioxidant-induced apoptosis, and modulation of key pathways such as phosphoinositide-3 kinase/RAC-alpha serine/threonine-protein kinase (PI3K/Akt) and mitogen-activated protein kinase (MAPK). These effects are especially notable in the prevention and progression of breast, colon, liver, and lung cancers, with statistical significance (*p* < 0.05). We highlight the role of flavonoids in modulating cell-signaling pathways, including the inhibition of the nuclear factor kappa-light-chain-enhancer of activated B cells (NF-kB) pathway and the promotion of apoptosis and autophagy in cancer cells.

Flavonoids, especially those found in fruits, play an important role in the food matrix and are attracting interest for their biosynthetic capabilities and optical properties, including fluorescence stability [8]. These flavonoids are not formed in the body, but their physiological functions affect the human body, and the mechanism of these effects is explained by the physiological functions of natural plant flavonoids such as quercetin, myricetin, rutin, and flavonoids of fruits [9]. Flavonoids are mostly found in yellow fruits such as pears, apples, plums, etc. [10]. Dietary flavonoids, such as quercetin, have emerged as promising anticancer candidates and are in clinical trials. Consumption of flavonoid-rich fruits has shown promising effects against certain cancers, particularly breast, colon, liver, and lung cancer [11]. Flavonoids prevent cancer through modulation of PI3K/Akt and MAPK pathways in breast cancer and exert anti-inflammatory effects and participate in antioxidant-induced apoptosis, fighting colon cancer [12]. In liver cancer, they are associated with antiangiogenic and antiproliferative effects, and in lung cancer, its mechanism of action operates through inhibition of the NF-kB pathway [13]. Positive correlations have been found for prostate, colon, and breast cancer; additionally, the scavenging of reactive oxygen species (ROS) and the induction of circulating apoptosis and autophagy suggest a role as a cancer-cell antioxidant and a positive modulator of pro-inflammatory cytokine signaling pathways [14].

Flavonoids have been reported to combat cancer through a mechanism of angiogenesis regulation [15]. The therapeutic properties of flavonoids are mediated through the modulation of cell-signaling pathways found downstream of PI3K tyrosine kinase growth receptor. They also have an opposite effect on signaling downstream of protein kinase B (Akt), a protein mutant effector protein of protein kinase A [16,17]. Flavonoids are involved in the modulation of Akt/PI3K/mammalian target of rapamycin (mTOR) pathway that stimulates and regulate the growth and development of cancer cells. They block PI3K activity and block crosstalk between PKB and PI3K, among other key proteins, including Akt, phosphatase and tensin homolog (PTEN), extracellular-signal-regulated kinase (ERK), and several others [18,19]. This effect attenuates H_2_O_2_-induced oxidative damage and induction of apoptosis in Leydig cells by the PI3K/Akt signaling pathway [20,21,22]. In addition, the adenosine triphosphate (PI3K-ATP) binding domain interferes with the mechanical target of the rapamycin complex 2 (mTORC2) complex to prevent Akt phosphorylation and induce antioxidant activity [23]. In response, flavonoids regulate the expression of various molecules, including glycogen synthase kinase-3 (GSK-3), and are involved in the promotion of TNF-α-induced apoptosis of human lung cancer cells [24]. The anticancer role of flavonoids is based on their role in angiogenesis through the expression of vascular endothelial growth factor (VEGF) [25,26]. They inhibit the activity of signal transducer and activator of transcription 3 (STAT3) by inhibiting proto-oncogene tyrosine-protein kinase Src kinase phosphorylation, which inhibits the growth and metastasis of cancer cells. STAT signaling is a characteristic signaling pathway in cancer, indicating a potential anticancer role for flavonoids [27,28,29].

**Figure 1:**
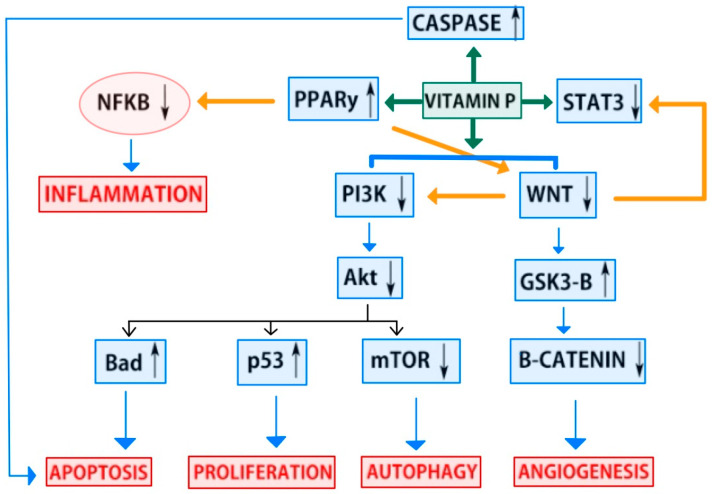
Mechanisms involved in the anticancer potential of flavonoids through the targeting of numerous molecular signaling pathways. Flavonoids inhibit cancer growth and differentiation by modulating key signaling pathways, including STAT5, STAT3, and JAK2. These pathways are crucial in cell growth, survival, and inflammatory responses. Overactivation of STAT3 and STAT5 is linked to cancer progression, while JAK2 activation is essential for STAT protein function in the JAK-STAT pathway.

Figure 1 depicts STAT5, STAT3, and Janus kinase 2 (JAK2) signaling acting to prevent the growth and differentiation of cancer cells. This systematic review therefore investigates the effect of these pathways on cancer prevention in the context of flavonoids derived from a healthy plant-based diet. We focus on the current literature on the role of flavonoid-involved signaling pathways. The STAT5, STAT3, and JAK2 signaling pathways play an important role in inhibiting the growth and differentiation of cancer cells. STAT proteins are key components of intracellular signaling pathways and are involved in cell growth, survival, differentiation, and inflammatory responses. In particular, STAT3 and STAT5 are overexpressed or overactivated in several types of cancer and are known to promote cancer progression and malignancy. JAK2 is an enzyme that is responsible for activating STAT proteins, making it an important part of the JAK-STAT pathway. This pathway involves binding to cytokine receptors on the cell surface to activate JAK2, which in turn phosphorylates and activates STAT proteins. Activated STAT proteins affect the growth and survival of cells by traveling to the nucleus and promoting the transcription of certain genes.

By elucidating these specific mechanisms and pathways, this study contributes to a comprehensive understanding of flavonoids’ role in cancer prevention. The findings underscore the potential for developing natural anti-cancer therapeutics through the inclusion of flavonoid-rich fruits in the diet. Future research should focus on randomized controlled trials assessing the long-term effects of flavonoid supplementation, exploring optimal dosages, and understanding interactions with conventional cancer therapies to provide comprehensive evidence for clinical applications.

## 2. Methods

**Figure 2:**
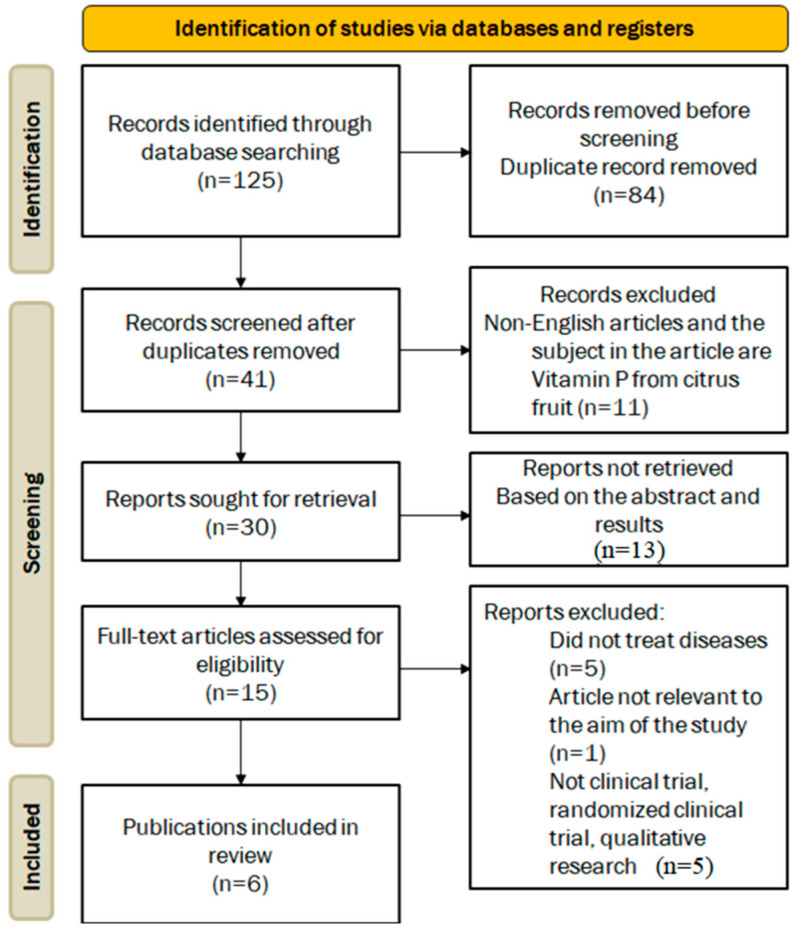
PRISMA flow diagram.

Although this review was a narrative literature review, we searched PubMed, Medline, Web of Science, Embase, SCOPUS, and Google Scholar according to PRISMA guidelines, and the search-term chain was: “flavonoid”, “flavonoid compound”, “flavonoid subclass”, “flavonoid-rich”, “fruit”, “citrus fruit”, “berry”, “cancer prevention”, “anticancer”, “chemoprevention”, “cancer risk”, “reduction”, “health benefit”, “health effect”, “disease prevention”, “wellness”, “antioxidant”, “antioxidant activity”, “reactive oxygen species”, “free radical scavenging”, “dietary intake”, “nutrition”, “food source”, “flavonoid and health”, “signal transducer and activator of transcription”, “STAT”, and “nutrient”. Figure 2 shows a flowchart of the process involved in finding and selecting the studies included in this literature review. In addition, the search for recent publications (published from 2020 to 2024) included specific keywords such as “epidemiology”, “population study”, “cohort study”, “case-control study”, “clinical study”, “human trial”, “clinical trial”, and “intervention study”.

### 2.1. Eligibility Criteria

Studies included in this review had to meet the eligibility criteria for inclusion. Literature-review-relevant clinical case studies, randomized controlled trials, experimental studies, longitudinal studies, epidemiologic studies, and bioactivity studies related to flavonoids were included, while review articles, case analyses, theses, letters, editorials, articles without full text, and clinically problematic articles were excluded.

### 2.2. Screening and Data Extraction

Inclusion criteria required that studies be original research articles, review articles, internet articles, letters to the editor, editorials, opinions, protocols, perspectives, commentaries, short communications, or position papers. There were no publication-date or language restrictions for articles, reports, briefs, and series. Articles that did not contain raw data in accessible full text, that were of inappropriate status, that pertained to inappropriate subject matter, or that were irrelevant to the main focus of the article were excluded.

### 2.3. Study Selection and Data Extraction

The data-extraction process involved two independent reviewers examining the titles and abstracts of all included studies, selecting the most relevant studies, and reviewing the full text to extract the required data. A standardized form was used for data extraction, and any discrepancies were resolved by a third reviewer. We assessed the quality and risk of bias of the included studies using the Cochrane Collaboration’s Risk of Bias tool and the Newcastle−Ottawa Scale (NOS). The criteria included study design, sample size, data collection methods, clarity of results reporting, and statistical analysis methods. The Cochrane Risk of Bias tool evaluated domains such as random sequence generation, allocation concealment, blinding of outcome assessment, incomplete outcome data, selective reporting, and other biases. Each domain was rated as “low risk”, “high risk”, or “uncertain risk”. This systematic assessment determined the overall risk of bias for each study included in our review. The NOS scale, which assesses selection, comparability, and outcomes, has a maximum score of 9 and reflects the methodology, quality, and potential risk of bias in the study design. The results are summarized in Table 1 to highlight the strengths and weaknesses of the studies. Inclusion criteria required that publications be studies on natural flavonoids from fruits; studies conducted in animals or humans; original experimental studies published in English; and studies examining the effects of flavonoids on human health, lung health, and cancer prevention. Excluded were conference abstracts, single-presentation studies, and abstracts presented at conferences or seminars. From the initial 125 identified references, 84 duplicates were removed, leaving 41 unique articles. After rigorous evaluation, five articles met the inclusion criteria and were selected for detailed analysis. However, caution is warranted due to the varying risk of bias observed across the included studies. Sensitivity analyses reaffirmed the consistency of results across most conditions despite inconsistencies stemming from study design and participant characteristics synthesized through meta-analysis.

## 3. Dietary Sources of Flavonoid

Flavonoids can be obtained through the consumption of tomatoes, mulberries, Amazon grapes, apples, and citrus fruits, as shown in Table 1 [30,31,32,33,34]. Flavonoid-rich fruits and vegetables have been shown to be positively protective against colon, prostate, and breast cancer and may have protective effects as chemopreventive agents. Flavonoids interact with proteins through dietary intake, raising the possibility of using synthetic analogs as anticancer therapeutics [35]. Tomatoes provide flavonoids and phytochemicals, which are biosynthetic compounds that may protect against various types of cancer [36]. Phenolic compounds, flavonoids and carotenoids, vitamins, and tomatins (glycoalkaloids) have been identified as phytochemicals that are particularly beneficial in the prevention of chronic degenerative diseases and are widely recognized for their cancer-fighting properties [37]. Specific flavonoids in tomatoes have antiproliferative activity, inducing different types of apoptosis [38]. In addition, tomatoes contain quercetin3-β-D-glucoside, which has antiproliferative properties and has been proven to be safe and stable, providing a potent antiproliferative effect on cancer cell lines [39,40]. Quercetin and flavonoids provide the most growth inhibition with the least cytotoxicity against colon (HT-29 and HCT 116), breast (MCF-7), hepatocellular (HepG2), and lung cancer (A549) cells [41]. The flavonoids and polyphenols in tomatoes have been shown to be related to several transcription factors and the conversion of phenylalanine to trans-cinnamic acid by phenylalanine ammonia lyase (PAL), which in turn catalyzes the conversion of cinnamic acid 4-hydroxylase (C4H) to p-coumaric acid. This process occurs due to the spatial accumulation of polyphenols in the fruit and the utilization of the amino acid phenylalanine in the shikimate pathway via the phenylpropanoid biosynthetic pathway [42,43].

Mulberry is a nutritious food that has long been used in folk medicine for its antioxidant properties [44]. In particular, the polyphenolic compounds isoquercitrin, chlorogenic acid, quercetin, astragalin, and kaempferol, along with flavonoids, may attenuate obesity-related inflammation and attenuate type 2 diabetes [45]. Mulberry also has anti-cancer properties against breast, liver, colon, and lung cancer cells and is involved in apoptosis as a factor of PI3K, tumor protein p53, c-Jun N-terminal kinase, and nuclear factor-kappaB (NF-kB) [46,47]. Additionally, mulberry flavonoids have proven positive therapeutic effects against numerous diseases, including antioxidant, antidiabetic, antihyperlipidemic, and anti-obesity effects [48]. Chlorogenic acid from the leaves has been shown to help prevent steatohepatitis through a reduction in oxidative stress [49]. It also scavenges excess oxygen radicals produced by HepG2 cells in response to oxidative stress; prolongs the lifespan of HepG2 cells by regulating the nuclear factor erythroid 2-related factor 2 (Nrf-2) signaling pathway; and reduces inflammation by decreasing the expression of TNF-α, interleukin 6 (IL-6), inducible nitric oxide synthase (iNOS), and nuclear factor kappa-light-chain-enhancer of activated B cells (NF-κB) [50].

Phenolic compounds in Amazon grape have potentiating effects on several sulfur oxidizing enzymes, including glutathione and superoxide dismutase (SOD), and are associated with the expression and activation of catalase and other detoxification and antioxidant enzymes [51,52,53]. They are involved in triggering the apoptotic process in chronic degenerative diseases such as diabetes, cardiovascular disease, and various cancers due to their effects [54,55]. Amazon grape is also an excellent source of polyphenols, which have very powerful disease-preventing properties and have been shown to improve human health. It has health benefits against DNA damage, protein damage, unstable iron activity, and enzyme inhibition and has also been shown to inhibit the in vitro activities of AChE, tyrosinase, and α-amylase, suggesting its potential as a preventive treatment for disease [56]. The cancer chemopreventive effects of these grapes are attributed to flavonoids, as well as to raspberatrol, quercetin, kaempferol, catechins, epicatechins, and anthocyanins (cyanidins and malvidin), which underlie their chemopreventive and antiproliferative effects [57].

Along with flavonoids, apples are a major source of bioactive compounds such as phenols, flavonoids, terpenoids, and carotenoids, which play a role in reducing cancer risk [58,59]. These fruits are rich in flavonoids, each of which has a variety of anti-cancer and health-promoting properties. In particular, the flavonoids they contain have been clinically proven to have antioxidant, anti-inflammatory, and anti-cancer bioactive effects. Polyphenols in apples, including chlorogenic acid, epicatechin, caffeic acid, coumaric acid, quercetin-3-glucoside (Q3G), quercetin, phlorizin, and phloretin, are particularly effective in preventing cardiovascular disease, diabetes, and cancer through phytochemical action [60]. In addition to quercetin, flavonoids in apples exhibit strong antioxidant activity due to the presence of polyphenols, flavonoids, and various phytochemicals [61]. The amount of active ingredients is determined immediately after harvest due to potential losses during warehousing and storage changes [62].

Flavonoids and nobiletin, which are central to flavonoid changes and antioxidant solubility, decrease with fruit growth and development, as do a number of biologically active compounds in citrus fruits [63]. Polyphenols are found in various parts of citrus fruits, including the skin, peels, seeds, pulp membrane, and juice, and their flavonoids have been shown to have antiviral, antifungal, and antibacterial activity [64]. Flavonoids are present especially in many orange varieties and are present at especially high levels in grapefruit. Additionally, fruits such as sweet oranges, mandarin oranges, and lemons contain relatively small amounts of flavonoids, which are used as a dietary antioxidant, especially in Asian countries, where they are extracted in conjunction with the hesperidin component of oranges, mandarins, and grapefruit [64,65]. Additionally, the useful flavonoid glycosides in citrus fruits are known as flavonoids, and these combine with hydrogen peroxide to inhibit radical activity and protect against cellular damage. They play an important role in anti-cancer activity and are especially effective in protecting capillaries, reducing hemophilia symptoms, and relieving leg edema [66,67]. Citrus flavonoids also have cytotoxicity-reducing effects, reducing the risk of heart disease from oxidized low-density lipoprotein (LDL) and cholesterol and protecting cell walls [68]. Flavonoids from citrus fruits such as pomelo, lime, lemon, mandarin, and grapefruit are widely distributed in aromatic plants and are found in particularly high concentrations in their peels [69]. These citrus-peel flavonoid compounds are useful in cancer treatment, with strong preclinical anti-cancer mechanisms identified, including radical scavenging [70]. Sweet cherries are also an antioxidant-active fruit that contain flavonoids with anti-cancer properties and can be an effective supplement for prevention of cardiovascular disease, obesity, diabetes, and degenerative diseases. This is due to a variety of bioactive components, including flavonoids, which are natural antioxidants that are stable and bioavailable [71]. Hackberry is a fruit native to the Mediterranean region and has high antioxidant capacity based on phenols and flavonoids. It has antibacterial properties due to the flavonoids, and the flavonoid content varies depending on the climate and on the origin. Its anti-bacterial properties have a high correlation with chlorogenic acid levels [72]. However, the biological therapeutic potential of some of the flavonoids in fruits is determined by their type, bioavailability, and possible mode of action, so their pharmacological utilization in clinical trials and epidemiological studies is important [73].

**Table 1:** Fruits that serve as dietary sources of flavonoids.

Dietary Source	Vitamin P Content	Anticancer Properties	Reference
Tomato	Average content of 20 mg 100 g^−1^ 14–20 mg 100 g^−1^ on a dry basis	Flavonoids, lycopene and carotenoids protect against cellular damage and prevent prostate cancer and cardiovascular disease	[30]
Mulberry fruit	Average content is 0.14 ± 0.050% DW	Strengthens capillaries, reduces high blood pressure and decreases vascular permeability, has an anti-edema effect, reduces the risk of atherosclerosis, has antioxidant activity	[31]
Amazon grape	The rutin content in the peel is 155.45 ± 2.06 mg kg^−1^ Flesh 2.64 ± 1.21 mg kg^−1^ fresh weight	Cytotoxic effects in cancer cell lines	[32]
Apple	12.136~483.89 µg/g	Antioxidant activity and activity similar to that of ascorbic acid	[33]
Citrus fruits	326.59 mg/100 g	Health-promoting effects of citrus-fruit extracts (grapefruit extract, pomelo extract, naringin, etc.) and flavonoid biological systems	[34]
Sweet cherry	1092.56 μg/g (MAE) 646.03 μg/g (CSE)	Various bioactive compounds, rutin, quercetin, quinic acid, and kaemferol-3-rutinoside Suppresses cardiovascular disease, diabetes, degenerative diseases, and cancer	[71]
Hackberry	0.55 µg/g	Possesses phenolic compounds in addition to flavonoids, which have antibacterial and antifungal properties	[72]

MAE, microwave-assisted extraction; CSE, conventional solvent extraction.

## 4. Flavonoid and Apoptosis Promotion

### 4.1. Chemical Structure of Flavonoids

**Figure 3:**
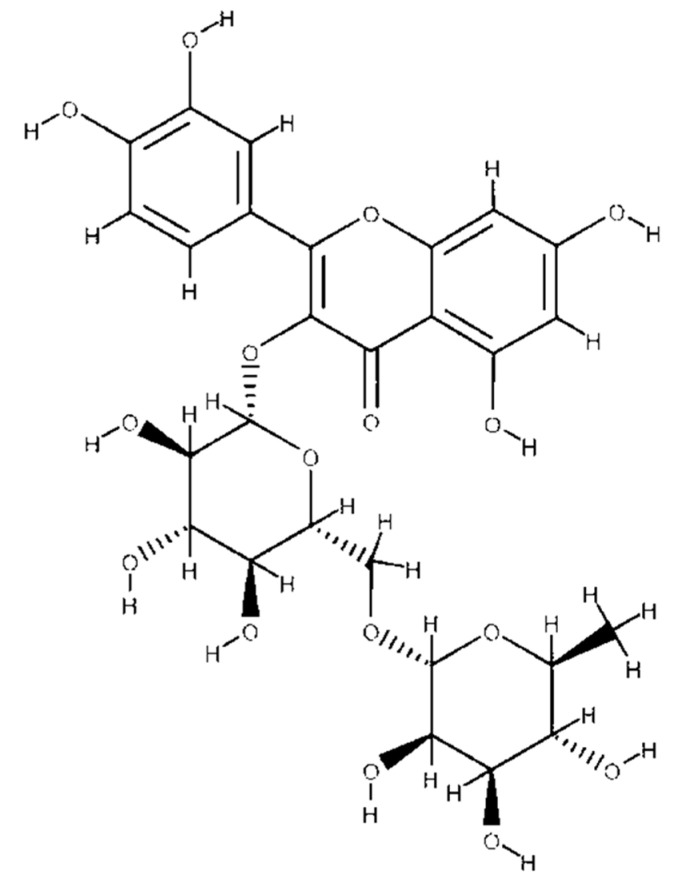
Chemical structure of a flavonoid.

Flavonoids are low-molecular-weight polyphenolic compounds found in more than 70 foods like grapefruit, cherries, apricots, buckwheat seeds, grapes, plums, and oranges. These compounds, known as citrus flavonoids, exhibit antioxidant, antibacterial, antiviral, and anti-inflammatory activities due to their hydroxyl groups [74]. They are also known as rutosides and sophorins and exhibit therapeutic activities such as antioxidant, antibacterial, antiviral, and anti-inflammatory activity due to the presence of hydroxyl groups [75,76]. The chemical structure of a flavonoid is shown in Figure 3.

Flavonoids act as plant-derived antioxidants with free-radical-scavenging properties, aiding in wound healing and supporting cell viability [77]. They are synthesized via the phenylpropanoid metabolic pathway, which involves converting phenylalanine to 4-coumaroyl-CoA and then to flavonoid structures through enzymatic modifications [78].

### 4.2. Anticancer Pathways of Flavonoids

Signaling pathways underlying molecular mechanisms of flavonoid activity are associated with strong activity of PI3K/Akt/GSK3β/NF-κB and p38/MK2 in colitis. Reduced expression of proinflammatory markers such as IgM, IgE, iNOS, ICAM-1, HO-1, and Th1/IL-10 cytokine ratios, as well as effects on effector modulation, regulation, and B cell homeostasis, represent a novel approach to treating colitis [79].

Flavonoids modulate several cell-signaling pathways, including PI3K/Akt, JAK/STAT, MAPK, and NF-κB, to exert anticancer effects [80]. They reduce inflammation and oxidative stress, inhibit platelet aggregation, and improve insulin sensitivity and lipid profiles, benefiting conditions like diabetes, hyperlipidemia, and cardiovascular disease [81,82]. Additionally, ROS-responsive nucleotide-binding domain-like receptor 3 (NLRP3) reverses endothelial dysfunction by enhancing and inhibiting nitric oxide production, which is in turn reduced by inflammasomes, thereby mitigating the risk of cardiovascular disease. Rutin has also been reported to counter neurodegenerative diseases by preventing neuroinflammation and abnormal protein accumulation and regulating cell death and microglial and astrocyte activation [83,84,85]. It has been reported to exert neuroprotective effects against various neurotoxins such as cisplatin, vancomycin, and mercury chloride through anti-inflammatory, antioxidant, anti-apoptotic, and aquaporin 1 level enhancement [86,87,88]. From another mechanistic perspective, rutin targets various inflammatory mediators such as NF-B-type κ and counteract TNF-α-induced inflammatory diseases. Hepatoprotective properties seen in animal models of nonalcoholic fatty-liver disease include abolishing key autophagy biomarkers and mitigating autophagy by regulating lipolysis and fat-synthesis genes [89]. In various preclinical models, flavonoids have also been shown to increase Nrf2 and to enhance the activities of enzymatic and non-enzymatic antioxidants such as SOD, CAT, and GPx, which may alleviate the aforementioned diseases [25,90].

Flavonoids may have a role in protection against dyslipidemia and breast cancer progression [91]. Elevated cholesterol levels are linked to uncontrolled cell growth and worse breast cancer prognosis [92]. Upregulation of FAS contributes to tumorigenesis by inhibiting apoptosis [93]. Specifically, flavonoids can inhibit cancer-cell growth by interfering with apoptosis and cell-cycle pathways. For example, they induce cell-cycle arrest in the G2/M phase and enhance apoptosis in breast cancer cells (MCF-7) by modulating the p53 and PTEN pathways [25,94,95]. They also counteract neurodegenerative diseases by preventing neuroinflammation and regulating cell-death mechanisms. Upregulation of p53 promotes activation of p21, leading to inhibition of numerous cell-cycle proteins, including CDK6, CDK2, CDK4, and cyclin B1 [96,97]. Flavonoids have been shown to modulate multiple signaling pathways involving Ras/Raf, PI3K/Akt, MAPK, TGF-β2/Smad2/3Akt/PTEN, either alone or in combination with other therapies. Additionally, flavonoids have been shown to enhance the effectiveness of chemotherapy drugs, reduce drug resistance, and mitigate chemotherapy-related side effects by synergistically inducing apoptosis in cancer cells [98].

## 5. Flavonoid-Rich Fruits

The flavonoids considered in this review include only natural flavonoids from fruits, which include compounds that are biosynthesized by a particular fruit (Table 2). The included methods involve quantitatively analyzing the amount of the compound in question in a sample of fruit. Clinically problematic articles include studies that have small sample sizes, use incorrect statistical analyses, or report results that have not been replicated in other studies. A Canadian case-control study showed that flavonoid intake had a favorable effect on lung cancer risk, and a Spanish case-control study showed that flavonoid intake may reduce the risk of colorectal cancer. The protective effect of flavonoid intake against breast cancer was particularly strong in Asian women and even stronger in postmenopausal women (OR 0.46, 95% CI 0.28–0.78). These findings show evidence from epidemiologic studies of flavonoid intake [99]. Dietary modification through fruit intake is essential for improving and positively impacting the prognosis of bladder cancer. A clinical trial involving 49 patients with non-muscle-invasive bladder cancer (NMIBC) demonstrated therapeutic benefits from a diet including *Cruciferae* [100]. The Mediterranean diet has been proven to reduce breast cancer recurrence [101]. Flavonoids naturally biosynthesized by tomatoes exhibit beneficial effects such as anti-oxidant activity. Additionally, the cleavage effect of flavonoids in tomatoes potentially leads to higher concentrations of secondary compounds such as polyphenols, lycopene, β-carotene, and lutein, which may enhance antioxidant capacity [102]. Dietary effects of tomatoes are also protective against prostate cancer [103]. Daily changes in fruit intake have demonstrated efficacy in preventing breast cancer [104], and clinical trials highlight the anti-atherosclerotic and anti-hyperglycemic effects of prickly pear (PP) fruit. PP juice consumption in healthy men positively affects heart-rate variability (HRV), total cholesterol (LDL-C, HDL-C), and glucose levels [105]. For cancer survivors, particularly older individuals, increased comorbidity and risk for secondary cancers necessitate diets rich in vegetables and fruits to improve overall health [106]. However, the clinical use of flavonoids must take into account their bioavailability, drug−drug interactions, and metabolic instability through isolation and purification from natural sources; mechanistic studies are also needed, as the flavonoids may be cytotoxic in their anticancer mechanisms [107].

## 6. Conclusions

The consumption of flavonoid-rich fruits is proposed as a potential lifestyle modification for cancer prevention, targeting angiogenic and cancer-protective pathways. However, it is important to note that while fruits rich in flavonoids also contain other phytochemicals that can benefit human health, the anti-cancer mechanisms of flavonoids remain underrepresented and poorly defined in the scientific literature. Our review highlights findings that supplementation with fruit products like citrus fruits (tomatoes, apples, grapes, and grapefruit) may induce angiogenic effects, anti-inflammatory actions, and antioxidant-induced apoptosis and may modulate pathways such as PI3K/Akt, MAPK, and TGF-β2/Smad2/3Akt/PTEN that are involved in anti-cancer responses. Future clinical trials focusing on flavonoids from fruits and vegetables, particularly on flavonoids from fruits, need to comprehensively address these mechanisms. Additionally, exploring the synergistic effects among various active ingredients and flavonoids in fruit foods, as well as examining multiple flavonoid compounds with anti-cancer potential, could serve as a foundation for developing natural anti-cancer therapeutics. However, it is essential to acknowledge the limitations and potential biases inherent in the reviewed studies to ensure a balanced and credible assessment of the findings.

To strengthen future research directions, it is crucial to focus on specific areas identified in this review. Firstly, conduct randomized controlled trials (RCTs) with larger, diverse populations to provide robust evidence on the efficacy of using flavonoid-rich fruits in cancer prevention. Secondly, prioritize mechanistic studies to elucidate how flavonoids inhibit angiogenesis and modulate pathways like PI3K/Akt and MAPK to exert anti-cancer effects. Thirdly, explore synergistic effects between flavonoids and other bioactive compounds in fruits for enhanced cancer prevention. Lastly, conduct longitudinal studies to assess the long-term dietary intake of flavonoids and its impact on cancer incidence and progression. Addressing these areas will advance our understanding and guide targeted clinical interventions in cancer prevention. Also, the conclusions would benefit from providing practical recommendations for clinicians and researchers. These recommendations should include specific guidance on incorporating flavonoid-rich fruits into dietary recommendations for cancer prevention. Additionally, suggesting targeted designs for clinical trials and mechanistic studies could fill existing research gaps effectively. These actionable insights can enhance studies’ relevance and impact in both clinical practice and further research endeavors.

## Figures and Tables

**Table 2:** Clinical trials of fruits rich in flavonoids.

Subjects	Target Cancer	Setting	Findings	Reference
49 NMIBC patients	Bladder cancer	Two-arm, double-blinded, randomized controlled trial A six-month intervention Daily consumption of fruits and vegetables	Significantly increased cruciferae intake and urinary ITC levels in NMIBC Survivors Improved bladder cancer outcomes and prognosis	[99]
1542 patients with breast cancer	Breast cancer	Randomized controlled trial Randomly assigned to an active dietary intervention	5 years of follow-up; 95 patients in the IG and 98 in the CG developed breast cancer recurrence. Self-reported diet at year 1 combined with IG and CG showed association of protection with higher degree of dietary change	[100]
79 patients with prostate cancer	Prostate cancer	Randomized to a nutritional intervention Controlled diet containing tomato products for 3 weeks.	A three-week nutritional intervention with tomato products alone or in combination with selenium and n-3 fatty acids lowers PSA in patients with metastatic prostate cancer.	[102]
1034 participants	Breast cancer	Two-arm randomized controlled trials	Similar long-term lifestyle influences include smoking, alcohol intake, dietary intake of fruit/vegetables/meat/vitamins, exercise, participation in breast cancer screening and reduced perception of cancer risk.	[103]
17 male patients	Cardiovascular disease	Double-blind, randomized, placebo-controlled, crossover trial	May moderate traditional responses to cardiovascular risk Nutrient content is important	[104]
731 medicare-eligible cancer survivors	Cancer prevention	A cross-sectional secondary analysis Kruskal−Wallis rank sum and post-hoc tests	Eating vegetables and fruits extends the life of cancer survivors. Gender, race, and cancer type affect survival.	[105]

NMIBC, non-muscle-invasive bladder cancer: IG, intervention group: CG, control group: PSA, prostate cancer.

## Data Availability

No new data were created or analyzed in this study. Data sharing is not applicable to this article.

## Abbreviations

PI3K—phospholnositide-3 kinase; Akt—protein kinase B; ERK—extracellular signal-regulated kinase; PTEN—phosphatase and tensin homolog; PI3K-ATP—adenosine triphosphate; mTORC2—mechanistic target of rapamycin complex 2; GSK-3—synthase kinase-3; VEGF—vascular endothelial growth factor; STAT 3—signal transducers and activators of transcription 3; ROS—reactive oxygen species; PAL—phenylalanine ammonia lyase; C4H—cinnamic acid 4-hydroxylase; NF-kB—nuclear factor-kappaB; Nrf-2—nuclear factor erythroid 2-related factor 2; IL-6—interleukin 6; iNOS—inducible nitric oxide synthase; Q3G—quercetin-3-glucoside; LDL—low-density lipoprotein; PR—polylutein; NLRP3—nucleotide-binding domain-like receptor 3; NMIBC—non-muscle-invasive bladder cancer; PP—prickly pear; HRV—heart-rate variability; SRC—proto-oncogene tyrosine-protein kinase Src; mTOR—mammalian target of rapamycin.
